# Co-development, co-production and co-dissemination of scientific research: a case study to demonstrate mutual benefits

**DOI:** 10.1098/rsbl.2020.0699

**Published:** 2021-04-14

**Authors:** Lucy C. Woodall, Sheena Talma, Oliver Steeds, Paris Stefanoudis, Marie-May Jeremie-Muzungaile, Alain de Comarmond

**Affiliations:** ^1^ Department of Zoology, University of Oxford, Oxford, UK; ^2^ Nekton, Oxford, UK; ^3^ Ministry of Agriculture, Climate Change and Environment, Victoria, Seychelles

**Keywords:** partnership, marine, collaboration, deep sea, transdisciplinary

## Abstract

Inadequate and inequitable distribution of research capacity and resources limits both the opportunity for leadership and participation in science. It also results in biases of effort, poor and misinterpretation of global patterns and the availability of limited usable knowledge for current challenges. Increased participation in ocean research and decision-making is needed to account for many stressors and challenges. The current intergovernmental attention on the ocean (e.g. UN Decade of Ocean Science for Sustainable Development) and the development of technologies that permit exploration and accelerate exploitation suggest that it is timely to focus on the ocean and its stewardship. Employing the principles of co-development, co-production and co-dissemination, this paper uses a case study of a deep reef project in Seychelles to illustrate some activities that can be employed to magnify research outcomes and legacy. We provide examples that range from ministerial briefings and planning meetings to joint fieldwork, grant allocation and co-authoring outputs. These activities helped us to align priorities, promote authentic interactions and focus on equitable science. Finally, reflecting on our experiences, we acknowledge the benefits brought by respectful and long-term partnerships, the variety of activities needed to develop these and challenges of maintaining them. In the future, we also want to include more opportunities for regional peer-to-peer learning and technology transfer.

## 1. Introduction

Opportunity to access marine space and the benefits it conveys is not equal across the planet [1,2]. Research conducted in waters below 30 m is historically exclusive, meaning that a limited number of researchers have had access to the equipment and funds that permit the surveying, sampling or exploring of these habitats. For example, despite the deep sea (greater than 200 m) being part of the national waters of 70% of nations, only 17% of these have access to scientific assets appropriate for this research [3]. This is often because of the specialist, costly and scarce nature of equipment that has traditionally been used in this environment and the vessels required to access it. This disconnect between the need for research and the experience and resources to undertake these studies requires resolution [4]. This is further exacerbated by the phenomenon of ‘parachute science’, which is a model of practice where scientists undertake fieldwork in a nation different from their own, and once samples or data are collected, they leave with little or no further engagement with anyone from that nation [5,6]. Typically, this involves scientists from a wealthier nation visiting a country with a lower gross domestic product [7]. Not only is this approach ethically questionable, it neglects to appreciate the value of the representation, skills, knowledge and perspectives of the resident scientists, stakeholders and government. It, therefore, often results in important questions not being asked, data not being fully interpreted and knowledge not being applied [8]. Ultimately, the extensive barriers that exist to accessing deeper waters result in slower rates of scientific discoveries coupled with the reduced and limited impact of both findings and benefits to society. In large part, this is because the research questions are being set by a select few, the local context is not included in research conclusions and associated new knowledge is removed from those who need it the most and/or information is not presented in a usable form.

Initiatives for widening participation in research activities on mesophotic coral ecosystems (approx. 30–150 m) and the deep sea do exist, through equipment dissemination and training [9,10], providing exposure to deeper habitats through tele-presence [11], youth fellowships and training experiences [12] and programmes that facilitate scientists joining expeditions [13]. Together, these programmes demonstrate the highly variable activities that are being conducted to strengthen knowledge exchange and ocean literacy globally. However, we believe these initiatives are not incorporated widely enough to meet the needs of ocean nations. It is vital that meaningful engagement is prioritized with scientific, policy and civil society stakeholders to foster a relationship of trust and mutual skills sharing. This transdisciplinary approach takes time and resources, but we believe it produces pathways to impact and opportunities for robust science. Furthermore, science conducted in this manner provides a solid base from which to interpret global patterns and to highlight when these are constructed from biased information.

Transdisciplinary research is achieved by fully integrating stakeholders from academic and non-academic fields throughout the research process and is recognized in sustainability science [14]. Collaborative research is implicit for many scientists as a necessity to address research questions and to access equipment; however, there is a call for even greater integration to achieve more useful outcomes [15,16]. Co-developing projects and co-producing knowledge in scientific research and environmental management is not new. There have been many successful initiatives, for example, in ecosystem management in the Arctic [17], charismatic species research [18] and most extensively in small-scale fisheries management [19]. Yet despite obligations under the United Nations Convention for the Law of the Sea for research to be collaborative, inclusive of coastal states and incorporate knowledge transfer [20], co-development and co-production of knowledge are the exception not the rule.

This paper sets out to provide illustrative examples of activities that facilitated engagement and cooperation during marine research project in Seychelles which was a collaboration between national and international parties. The paper also identifies key reflections from our experience.

## 2. Project principles

A transdisciplinary research project requires the continuous involvement of and leadership from a range of stakeholders throughout its duration. This can only be achieved by placing the principles of co-development, co-production and co-dissemination right at the heart of the project.

These principles firstly highlight that a specific shared goal or challenge should underpin the project. Secondly, the co-development of the research agenda requires a range of different approaches to ensure equitable participation and engagement by stakeholders. Thirdly, knowledge co-production should be specific to the context of the situation, while recognizing there is no rule book for creating meaningful interactions. Finally, the new information gathered must be co-disseminated into outputs and products that are audience-appropriate. The transdisciplinary framework, therefore, serves to help create new knowledge for all, ensures that all stakeholders have ownership of the research and helps to promote legitimacy of the findings [21].

**Figure 1.**
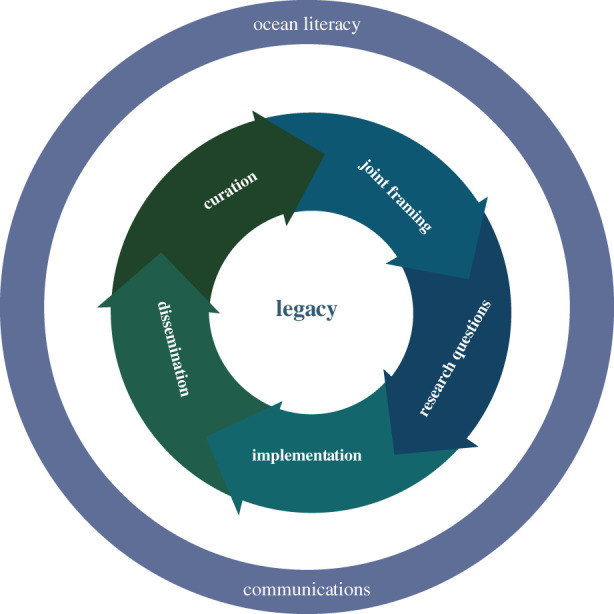
The transdisciplinary framework used to plan our marine research programme.

Our research process drew on the Transdisciplinary Future Earth framework [22]. At the core, the research process was its legacy, which was supported by communication and ocean literacy activities throughout (figure 1). The process started with jointly framing the research agenda with an extensive range of stakeholders (‘joint framing’, figure 1). Subsequently, this agenda was translated into specific research questions that were of interest to the different partners (‘research definition’, figure 1). The agreed research was then jointly planned and conducted, while data and results were analysed and interpreted collaboratively (‘implementation’, figure 1). New findings were then disseminated across partners and stakeholders in a parallel manner and with a specific focus on audience needs (‘dissemination’, figure 1). Finally, collected samples and data, together with any generated knowledge outputs and products, were appropriately curated (‘curation’, figure 1).

## 3. Case study

### (a) The context

Seychelles is an Indian Ocean island state of 1.37 million km^2^ committed to a marine spatial planning (MSP) process and to protecting at least 30% of their Exclusive Economic Zone (EEZ). This target was achieved in March 2020 and saw more than 220 stakeholder engagements from 10 different committees and groups represented by over 75 people. The process was led by the Seychelles government and facilitated by a US non-governmental organization (NGO), The Nature Conservancy [23], and much of the shallow water data used were generated by a plethora of Seychelles-based NGOs, academics and government departments. The protected areas include regions of the deep sea despite very little specific data from these areas, and this knowledge gap was identified during the MSP process. It was also recognized that this gap was partly due to very limited access to equipment to document marine life in deeper waters and partly due to not being able to access all data/samples collected by previous projects. At the invitation of the Government of Seychelles, the UK NGO Nekton facilitated an expedition, conducted by Seychellois partners (who were coordinated by the Ministry of Agriculture, Climate Change and Environment (MACCE)) and international scientists, to provide data and conclusions about mesophotic and deeper reefs (30–250 m) across the Seychelles Outer Islands.

The fieldwork focused on exploring marine ecosystems across islands. This work integrated five specific projects led by Seychellois scientists (funding by the Nekton/Seychelles Conservation and Climate Adaptation Trust partnership), with broader questions on biotic and abiotic parameter patterns, and all on board took part in all activities. During the expedition, an ocean-going vessel was used to deploy the specialist research equipment that is not available in Seychelles (e.g. submersibles and multibeam sonar) and focal sites were prioritized based on a previously agreed list founded on academic literature, technical reports, local wisdom and knowledge needs that were identified during earlier consultative workshops.

The initial expedition report [24] was co-authored by all participants, and a benthic field identification guide is being co-written by a wider range of partners. Findings from the expedition are currently being considered by partners, which will result in additional manuscripts and policy briefing documents. These will help inform management plans for the newly protected areas and should increase our understanding of deep reefs globally.

### (b) Practical pathway

The team undertook specific activities before, during and after the fieldwork phase (https://nektonmission.org/missions/first-descent-seychelles). Presented activities (table 1) are examples of what was done; they are not intended to be a roadmap for projects in other nations with other partnerships, but rather provide concrete examples that were considered useful. By acknowledging historic and contemporary power balances and dynamics (e.g. Seychelles is a former British colony that became an independent republic in 1976), it was important to place the Seychelles partners at the centre of the decision-making and communications from the start. This also helped ensure enhanced engagement and interest from the resident population, who are crucial to ensure a long-lasting legacy of the project. We aimed to create shared goals that were needs-led and that resulted in activities and findings that were transparent and open. To do this, policy-makers and scientists alike were involved during all stages of the project. Goals and findings were communicated pluralistically depending on context in order to share benefits of the knowledge across team members.

**Table 1.** Phased approaches to co-developing, co-producing and co-disseminating marine research with exemplar activities. MOU, memorandum of understanding.

phase	objective	key concepts	example activities	communication and ocean literacy component
joint framing of the research agenda	with host nations, define the terms of reference of the research programme for both local and global benefits	– engagement from all levels and sectors	– gain political endorsement in country – establish partnerships and formalize with MOUs and grant partnership agreements – in-country meetings with decision-makers and stakeholders	– meet with head of state – ministerial briefing
define research questions and build a research team	pose, refine and prioritize research questions, with partners and stakeholders, determine leadership of projects and ensure the ownership of all	– define a pathway to impact, timeline and the actors and their roles, and formalize with memorandum of understanding or other appropriate mechanism	– science planning workshop – logistics workshop – bilateral phone calls and online meetings – grant programme established for host nation scientists – co-authored research permit and research plan	– local media interviews – international publicity of ocean issues (e.g. press releases and international media interviews) – map school curriculum to ocean literacy – local ocean ambassadors
implement fieldwork	conduct research and create visibility of marine habitats >30 m	– data collection for multiple questions using the pre-define prioritization – host country participants who were successful in the grants programme lead their own research while sharing in the collective aims	– pre-expedition meeting – collaborations to provide expertise required – on-board experience sharing by participants e.g. basic taxonomic skills, data management, sample preservation and equipment deployment – virtual learning through tele-presence	– launch event – local media interviews – international publicity of the project – policy advisors and decision-makers visit the vessel
dissemination of results	sharing new information in a relevant manner for audiences to create new knowledge through peer-reviewed literature and other outputs that summarize the findings for policy-makers	– continued engagement of all parties to detect significance of findings to agree focus of analysis	– agree on format and content of final report and other outputs – partner-designed skills training workshops – review initial data with team – include and embrace all contributions for publications – accessible data and results – communicate results and relevance appropriate for audience	– local media interviews – international publicity of the project – open access publications – collaborative symposium – support host nation scientists to attend and present at international conferences
curation of new attributes and legacy	in accordance with national and international legislation and agreements, store and catalogue outputs and findings	– galvanize opportunities	– scientific journal club for all project members to discuss contemporary published research – using specimens collected, a specific taxonomic workshop including regional knowledge transfer – curation of specimens with appropriate institutions, ideally in the host country or institution agreed by project team – ensure material transfer agreements are in place if international loans and storage are required – deposit data in open access databases (raw data are subject to veto from host nation)	– knowledge-sharing network – regional expert hubs

## 4. Reflections

### (a) Unexpected benefits

An open dialogue with stakeholders and government during the planning and permitting stages facilitated approval of amended field locations, necessitated by poor weather, to be granted during the expedition. This meant no time was wasted while at sea. Furthermore, a clear expectation of sample collection and updates during the expedition meant that Convention on International Trade in Endangered Species of Wild Fauna and Flora (CITES) inspections could be made on arrival back into port, expediting the export permits, and this helped to facilitate MACCE to fulfil regulatory requirements.

### (b) Legacy potential

Importantly, all data collected are owned by Seychelles which, when possible, will permit open access to data for both Seychellois and international scientists. This provides a new foundation of knowledge for Seychellois marine scientists to develop future research and grant applications upon. On request from Seychelles partners, the team facilitated specific skills training that included the use of baited remote underwater stereo video systems. While short-term skills workshops are useful, long-term training schemes are most beneficial, so partners are endeavouring to incorporate more mentorship options in the future. It is hoped that project activities and the role models they created will result in a legacy of ongoing interest in the ocean, scientific research that is applicable to management, and conservation decision-making. Specifically, they provided experience, data, tools, knowledge, networks and leadership opportunities that are transferable to other projects and disciplines for both local and international partners. Seychellois authors AdC and M-MJ-M commented that ‘Understanding the collaborative process was also a valuable legacy for Seychelles. This expedition and associated activities provided an indication of how programmes can be run and where opportunities might be available for those Seychellois researchers who are interested in ocean science. Following this work, standards for engagement and have now been established by MACCE’.

### (c) Challenges can occur, but a good process minimizes occurrence and disruption

It is not straight-forward to build trust; it takes time, skill and resources, particularly finance and personnel. In future, even more emphasis should be made to align timelines across parties. Initial alignments may not always remain throughout the research project, and effort and understanding is required by all to focus resources on shared goals and work towards these. The legal agreements around data-sharing and curation are important but time-consuming. MACCE now have a draft template on which to start these negotiations and Nekton has a better appreciation of the sensitivities around these documents. Building relationships requires a flexible approach, and they do not always succeed in their original form, but designating partnership personnel based in Seychelles for coordinating all national and international partner(s) was useful to maintain momentum. This was aided by effective leadership and commitment from all parties.

### (d) Increased regional peer-to-peer learning and technology transfer

In the future, when experience and equipment permit, the regional dimension of knowledge-sharing should become more apparent, with those from nearby nations sharing directly with each other. This will support skills transfer as training at sea will always be limited by berth numbers. Peer-to-peer learning provides opportunities for regional knowledge network, expert hubs and powerful role models for the next generation of scientists. However, the value of strong partnerships, as in the case study given here, is still considered high for developing additional skills. Reflection has also revealed that future projects should have a means of technology transfer (equipment and the ability to mobilize and deploy it), in order to facilitate additional research.

### (e) Clear communication is important but not easy

Although stakeholders were introduced to international partners by MACCE and regular stakeholder meetings and bilateral updates were undertaken, the team has learnt that further effort to check in with people at all levels of each partnership would have helped to keep expectations aligned and to quickly amend them when situations changed. A tailored approach to communications to match partners' expectations, wishes and commitments is essential.

### (f) Nothing is perfect but long-term partnerships are valuable

Experience of working together is useful in order to co-create more tailored experiences in the future. After the field phase, feedback was sought and provided from all partners. However, it is vital to undergo a more reflective and critical analysis of all parts of the project before starting another.

## 5. Conclusion

Our experience suggests that conducting research using a transdisciplinary philosophy amplifies benefits of research [25]. The process and activities of the case study were not perfect or novel, instead they have been presented to show how very different types of activities can be integrated. Projects dedicated to increasing authentic interactions and engagement result in new knowledge outcomes that meet the specific needs and priorities of more stakeholders (e.g. [26]). With knowledge assimilated and owned in the country where fieldwork was conducted, projects create foundations on which to achieve a legacy of impact. Our reflections align with those of others who have specifically considered effective science collaborations in small island states [27] and who have suggested strategies for maximizing effective research when working overseas [28]. However, we feel that, within many current funding and research valuation models, the additional time, engagement and activities that are necessary to undertake this approach are rarely accounted for (but see [29]). Until there is routine support for partnership building and the resources required, we believe that there will be barriers to effective international and transdisciplinary research that benefits the global community at large.

With the current focus on the ocean at the United Nations level and the increasing recognition of its value across society, it is time for action. To achieve the aims and targets of international agreements (e.g. of SDG 14), more people globally will have to be engaged with the ocean and shape research agendas to meet their national and regional priorities and commitments. The opportunity to increase contributions and leadership of diverse groups in ocean research and stewardship is essential. The onus must be on each of us in the marine research community to ensure that our practices promote equality and equity. This is the only way to help to secure a better-managed ocean and ultimately a healthier planet.

## Data accessibility

This article has no additional data.
